# *catena*-Poly[[di-μ-bromido-dicopper(I)]bis­[μ-η^2^,σ^1^-4-(2-allyl-2*H*-tetra­zol-5-yl)pyridine]]

**DOI:** 10.1107/S1600536808012439

**Published:** 2008-06-07

**Authors:** Wei Wang

**Affiliations:** a Ordered Matter Science Research Center, Southeast University, Nanjing 210096, People’s Republic of China

## Abstract

The title compound, [CuBr(C_9_H_9_N_5_)]_*n*_, prepared by the solvothermal treatment of CuBr with 4-(2-allyl-2*H*-tetra­zol-5-yl)pyridine, is a new homometallic Cu^I^–olefin coordination polymer in which dinuclear Cu_2_Br_2_ units are linked by the organic olefin ligand 4-(2-allyl-2*H*-tetra­zol-5-yl)pyridine, which acts as a bidentate ligand connecting two neighbouring Cu_2_Br_2_ units through the pyridine N atom and the double bond of the allyl group. The coordination of Cu(I) is slightly distorted tetrahedral.

## Related literature

For the solvothermal synthesis and related structures, see: Ye *et al.* (2005 ▶, 2007 ▶).
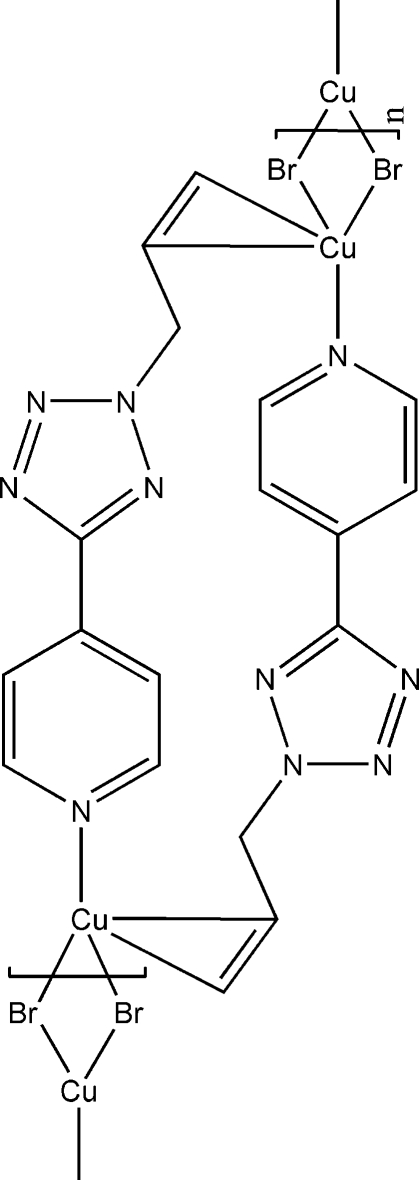


## Experimental

### 

#### Crystal data

[CuBr(C_9_H_9_N_5_)]
*M* *_r_* = 330.66
Monoclinic, 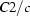

*a* = 17.502 (3) Å
*b* = 12.047 (2) Å
*c* = 13.664 (3) Å
β = 129.52 (3)°
*V* = 2222.4 (12) Å^3^
*Z* = 8
Mo *K*α radiation
μ = 5.54 mm^−1^
*T* = 293 (2) K
0.2 × 0.15 × 0.1 mm

#### Data collection

Rigaku Mercury2 diffractometer
Absorption correction: multi-scan (*CrystalClear*; Rigaku, 2005 ▶) *T*_min_ = 0.661, *T*_max_ = 1 (expected range = 0.380–0.575)
11222 measured reflections
2552 independent reflections
1962 reflections with *I* > 2σ(*I*)
*R*_int_ = 0.050

#### Refinement

*R*[*F* ^2^ > 2σ(*F* ^2^)] = 0.039
*wR*(*F* ^2^) = 0.088
*S* = 1.07
2552 reflections
145 parameters
H-atom parameters constrained
Δρ_max_ = 0.40 e Å^−3^
Δρ_min_ = −0.77 e Å^−3^

### 

Data collection: *CrystalClear* (Rigaku, 2005 ▶); cell refinement: *CrystalClear*; data reduction: *CrystalClear*; program(s) used to solve structure: *SHELXS97* (Sheldrick, 2008 ▶); program(s) used to refine structure: *SHELXL97* (Sheldrick, 2008 ▶); molecular graphics: *ORTEPIII* (Johnson & Burnett, 1997 ▶) and *ORTEP-3 for Windows* (Farrugia, 1997 ▶); software used to prepare material for publication: *SHELXL97*.

## Supplementary Material

Crystal structure: contains datablocks I, global. DOI: 10.1107/S1600536808012439/dn2345sup1.cif

Structure factors: contains datablocks I. DOI: 10.1107/S1600536808012439/dn2345Isup2.hkl

Additional supplementary materials:  crystallographic information; 3D view; checkCIF report

## Figures and Tables

**Fig. 1.**
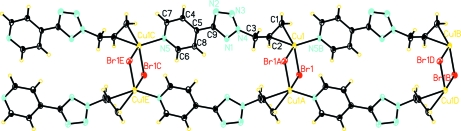
The one-dimensional structure of the title compound with the atom-labelling scheme. Displacement ellipsoids are drawn at the 30% probability level. H atoms have been omitted for clarity. [Symmetry codes: (i) 1 - x, y, 3/2 - z; (ii)x, y - 1, z; (C) x, y + 1, z; (iii) 1 - x, 1 + y, 3/2 - z.].

## supplementary crystallographic information

### Comment

Hydrothermal or solvothermal syntheses can offer some interesting reactions and compounds which can't be obtain through conventional solution techniques. In sealed tube, unstable copper (I) salt can exist under vacuums, and then interesting copper (I) organometallic compound can be prepared. The title compound is obtained through solvothermal treatment of CuBr and 4-(2-allyl-2*H*-tetrazol -5-yl) pyridine in methanol solvent at 75°C.

The copper(I) is coordinated to two organic ligands and two bridging Br atoms to fulfill its tetrahedral coordination environment (Fig 1).The organic ligand acts as a bidentate ligand connecting two neighbouring Cu_2_Br_2_ dinucler units through N atom from pyridine ring and double bond of the allyl group thus leading to an homometallic Cu^I^ olefin coordination polymer developing along the *b* axis. Unfortunately, the N atoms of the tetrazole ring fail to coordinate to Cu^I^.

### Experimental

A mixture of 4-(2-allyl-2*H*-tetrazol-5-yl) pyridine(20 mg, 0.2 mmol), CuBr (35 mg,0.4 mmol), and methanol (2 ml) sealed in a glass tube were maintained at 75 °C with yield 75%. Crystals suitable for X-ray analysis were obtained after 5 days

### Refinement

All H atoms were fixed geometrically and treated as riding with C—H = 0.93 Å (aromatic), 0.97 Å (methylene) or 0.98 Å (methine) with *U*_iso_(H) = 1.2*U*_eq _(C).

### Crystal data

**Table:**

[CuBr(C_9_H_9_N_5_)]	*F*(000) = 1296
*M**_r_* = 330.66	*D*_x_ = 1.977 Mg m^−^^3^
Monoclinic, *C*2/*c*	Mo *K*α radiation, λ = 0.71073 Å
Hall symbol: -C 2yc	Cell parameters from 10074 reflections
*a* = 17.502 (3) Å	θ = 3.0–28.8°
*b* = 12.047 (2) Å	µ = 5.54 mm^−^^1^
*c* = 13.664 (3) Å	*T* = 293 K
β = 129.52 (3)°	Block, colorless
*V* = 2222.4 (12) Å^3^	0.2 × 0.15 × 0.1 mm
*Z* = 8	

### Data collection

**Table:**

Rigaku Mercury2 diffractometer	2552 independent reflections
Radiation source: fine-focus sealed tube	1962 reflections with *I* > 2σ(*I*)
graphite	*R*_int_ = 0.050
Detector resolution: 13.6612 pixels mm^-1^	θ_max_ = 27.5°, θ_min_ = 3.0°
CCD_Profile_fitting scans	*h* = −22→22
Absorption correction: multi-scan (*CrystalClear*; Rigaku, 2005)	*k* = −15→15
*T*_min_ = 0.661, *T*_max_ = 1	*l* = −17→17
11222 measured reflections	

### Refinement

**Table:**

Refinement on *F*^2^	Primary atom site location: structure-invariant direct methods
Least-squares matrix: full	Secondary atom site location: difference Fourier map
*R*[*F*^2^ > 2σ(*F*^2^)] = 0.039	Hydrogen site location: inferred from neighbouring sites
*wR*(*F*^2^) = 0.088	H-atom parameters constrained
*S* = 1.07	*w* = 1/[σ^2^(*F*_o_^2^) + (0.03*P*)^2^ + 4.1679*P*] where *P* = (*F*_o_^2^ + 2*F*_c_^2^)/3
2552 reflections	(Δ/σ)_max_ = 0.001
145 parameters	Δρ_max_ = 0.40 e Å^−^^3^
0 restraints	Δρ_min_ = −0.77 e Å^−^^3^

### Special details

**Table:**

Geometry. All esds (except the esd in the dihedral angle between two l.s. planes) are estimated using the full covariance matrix. The cell esds are taken into account individually in the estimation of esds in distances, angles and torsion angles; correlations between esds in cell parameters are only used when they are defined by crystal symmetry. An approximate (isotropic) treatment of cell esds is used for estimating esds involving l.s. planes.
Refinement. Refinement of *F*^2^ against ALL reflections. The weighted *R*-factor *wR* and goodness of fit *S* are based on *F*^2^, conventional *R*-factors *R* are based on *F*, with *F* set to zero for negative *F*^2^. The threshold expression of *F*^2^ > σ(*F*^2^) is used only for calculating *R*-factors(gt) *etc*. and is not relevant to the choice of reflections for refinement. *R*-factors based on *F*^2^ are statistically about twice as large as those based on *F*, and *R*- factors based on ALL data will be even larger.

### Fractional atomic coordinates and isotropic or equivalent isotropic displacement parameters (Å^2^)

**Table:**

	*x*	*y*	*z*	*U*_iso_*/*U*_eq_	
Cu1	0.39530 (4)	−0.00789 (3)	0.59335 (4)	0.03898 (15)	
Br1	0.41669 (3)	−0.00735 (3)	0.79367 (3)	0.03492 (12)	
N1	0.4034 (2)	0.4106 (2)	0.5433 (3)	0.0379 (8)	
N2	0.3650 (3)	0.4582 (3)	0.3597 (3)	0.0448 (8)	
N3	0.3835 (3)	0.3508 (3)	0.3743 (3)	0.0475 (9)	
N4	0.4055 (2)	0.3250 (2)	0.4833 (3)	0.0363 (7)	
N5	0.3645 (2)	0.8317 (2)	0.5386 (3)	0.0319 (7)	
C1	0.2905 (3)	0.0849 (3)	0.4367 (4)	0.0397 (9)	
H1A	0.2909	0.0807	0.3661	0.048*	
H1B	0.2243	0.0836	0.4105	0.048*	
C2	0.3548 (3)	0.1574 (3)	0.5299 (4)	0.0376 (9)	
H2	0.3295	0.2001	0.5648	0.045*	
C3	0.4350 (3)	0.2120 (3)	0.5368 (4)	0.0449 (10)	
H3A	0.4950	0.2157	0.6246	0.054*	
H3B	0.4488	0.1681	0.4902	0.054*	
C4	0.3456 (3)	0.6920 (3)	0.4013 (3)	0.0356 (9)	
H4	0.3317	0.6742	0.3251	0.043*	
C5	0.3673 (2)	0.6090 (3)	0.4859 (3)	0.0285 (7)	
C6	0.3807 (3)	0.7513 (3)	0.6168 (3)	0.0338 (8)	
H6	0.3909	0.7711	0.6901	0.041*	
C7	0.3451 (3)	0.8007 (3)	0.4310 (3)	0.0366 (9)	
H7	0.3304	0.8554	0.3732	0.044*	
C8	0.3830 (3)	0.6403 (3)	0.5945 (3)	0.0338 (8)	
H8	0.3951	0.5871	0.6522	0.041*	
C9	0.3769 (3)	0.4926 (3)	0.4619 (3)	0.0315 (8)	

### Atomic displacement parameters (Å^2^)

**Table:**

	*U*^11^	*U*^22^	*U*^33^	*U*^12^	*U*^13^	*U*^23^
Cu1	0.0551 (3)	0.0158 (2)	0.0379 (3)	0.0008 (2)	0.0258 (2)	−0.00052 (17)
Br1	0.0433 (2)	0.0305 (2)	0.0396 (2)	−0.00627 (16)	0.03041 (19)	−0.00676 (15)
N1	0.0484 (19)	0.0190 (15)	0.0431 (19)	0.0017 (14)	0.0276 (17)	0.0017 (13)
N2	0.062 (2)	0.0236 (16)	0.043 (2)	0.0066 (16)	0.0307 (18)	0.0007 (14)
N3	0.068 (2)	0.0244 (17)	0.048 (2)	0.0036 (16)	0.036 (2)	−0.0030 (14)
N4	0.0409 (18)	0.0135 (14)	0.050 (2)	0.0011 (13)	0.0271 (17)	−0.0007 (13)
N5	0.0372 (17)	0.0154 (14)	0.0352 (17)	0.0002 (12)	0.0193 (15)	−0.0010 (12)
C1	0.039 (2)	0.0268 (19)	0.048 (2)	0.0042 (16)	0.0248 (19)	0.0102 (17)
C2	0.057 (2)	0.0164 (17)	0.047 (2)	0.0117 (17)	0.037 (2)	0.0068 (15)
C3	0.044 (2)	0.0163 (18)	0.064 (3)	0.0055 (16)	0.029 (2)	0.0065 (17)
C4	0.051 (2)	0.0209 (17)	0.031 (2)	−0.0029 (16)	0.0243 (19)	−0.0024 (14)
C5	0.0269 (17)	0.0171 (16)	0.0332 (18)	−0.0031 (14)	0.0153 (16)	−0.0011 (13)
C6	0.045 (2)	0.0199 (17)	0.038 (2)	−0.0036 (15)	0.0272 (19)	−0.0026 (15)
C7	0.049 (2)	0.0185 (17)	0.037 (2)	0.0002 (16)	0.0248 (19)	0.0047 (15)
C8	0.042 (2)	0.0199 (17)	0.036 (2)	−0.0025 (15)	0.0236 (18)	0.0034 (14)
C9	0.0322 (17)	0.0168 (17)	0.0372 (19)	−0.0029 (14)	0.0182 (16)	0.0003 (14)

### Geometric parameters (Å, °)

**Table:**

Cu1—N5^i^	2.017 (3)	C1—H1A	0.9700
Cu1—C1	2.050 (4)	C1—H1B	0.9700
Cu1—C2	2.106 (3)	C2—C3	1.496 (6)
Cu1—Br1	2.5156 (9)	C2—H2	0.9800
Cu1—Br1^ii^	2.5973 (11)	C3—H3A	0.9700
Br1—Cu1^ii^	2.5973 (11)	C3—H3B	0.9700
N1—C9	1.330 (4)	C4—C7	1.373 (5)
N1—N4	1.332 (4)	C4—C5	1.386 (5)
N2—N3	1.317 (4)	C4—H4	0.9300
N2—C9	1.340 (5)	C5—C8	1.378 (5)
N3—N4	1.317 (5)	C5—C9	1.475 (4)
N4—C3	1.475 (4)	C6—C8	1.378 (5)
N5—C6	1.332 (4)	C6—H6	0.9300
N5—C7	1.334 (4)	C7—H7	0.9300
N5—Cu1^iii^	2.017 (3)	C8—H8	0.9300
C1—C2	1.351 (5)		
			
N5^i^—Cu1—C1	106.55 (13)	C3—C2—Cu1	109.5 (2)
N5^i^—Cu1—C2	144.30 (14)	C1—C2—H2	115.7
C1—Cu1—C2	37.93 (14)	C3—C2—H2	115.7
N5^i^—Cu1—Br1	103.04 (9)	Cu1—C2—H2	115.7
C1—Cu1—Br1	123.33 (12)	N4—C3—C2	110.9 (3)
C2—Cu1—Br1	102.94 (10)	N4—C3—H3A	109.5
N5^i^—Cu1—Br1^ii^	99.28 (9)	C2—C3—H3A	109.5
C1—Cu1—Br1^ii^	124.96 (11)	N4—C3—H3B	109.5
C2—Cu1—Br1^ii^	102.07 (11)	C2—C3—H3B	109.5
Br1—Cu1—Br1^ii^	95.64 (4)	H3A—C3—H3B	108.0
Cu1—Br1—Cu1^ii^	84.36 (4)	C7—C4—C5	119.4 (3)
C9—N1—N4	101.0 (3)	C7—C4—H4	120.3
N3—N2—C9	106.6 (3)	C5—C4—H4	120.3
N4—N3—N2	105.6 (3)	C8—C5—C4	117.6 (3)
N3—N4—N1	114.3 (3)	C8—C5—C9	121.8 (3)
N3—N4—C3	122.5 (3)	C4—C5—C9	120.6 (3)
N1—N4—C3	123.2 (3)	N5—C6—C8	123.3 (3)
C6—N5—C7	117.1 (3)	N5—C6—H6	118.3
C6—N5—Cu1^iii^	121.9 (2)	C8—C6—H6	118.3
C7—N5—Cu1^iii^	119.8 (2)	N5—C7—C4	123.2 (3)
C2—C1—Cu1	73.3 (2)	N5—C7—H7	118.4
C2—C1—H1A	116.2	C4—C7—H7	118.4
Cu1—C1—H1A	116.2	C6—C8—C5	119.3 (3)
C2—C1—H1B	116.2	C6—C8—H8	120.3
Cu1—C1—H1B	116.2	C5—C8—H8	120.3
H1A—C1—H1B	113.2	N1—C9—N2	112.5 (3)
C1—C2—C3	122.0 (4)	N1—C9—C5	123.2 (3)
C1—C2—Cu1	68.8 (2)	N2—C9—C5	124.2 (3)

Symmetry codes: (i) *x*, *y*−1, *z*; (ii) −*x*+1, *y*, −*z*+3/2; (iii) *x*, *y*+1, *z*.
